# Supplementary material for: Geometric and quantum kernel methods for predicting skeletal muscle outcomes in experimental chronic obstructive pulmonary disease
Source: Sci Rep. 2026 Sep 10;16:28367. doi: 10.1038/s41598-026-65964-w (PMC13562544; doi:10.1038/s41598-026-65964-w)
Supplement: Supplementary file 1 [file 41598_2026_65964_MOESM1_ESM.pdf]

Supplementary Information

Repeated-CV reference tables and statistical tests

This Supplementary Information reports the complete repeated-CV results used for the main manuscript, including unreduced main and sensitivity tables, paired repeated-CV statistical tests, method-family descriptions, and the synthetic-medoid reference-selection audit. Main-text rankings use the repeated-CV results from the 15 held-out outer-fold evaluations.

Repeated-CV Main Tables

Table 1: Repeated 3×5 cross-validation main results for force readout.

Group	Model	CV folds	RMSE ↓	RMSE SD	MAE ↓	R^2 ↑	ROC–AUC ↑
**Classical**	**Biomarker-only Ridge**	**15**	**2358.459**	**233.420**	**1981.346**	**0.562**	**0.896**
Classical	LDA condition axis then Ridge engineered	15	2364.686	256.495	1987.384	0.560	0.888
Classical	Compact Ridge + condition	15	2365.346	232.737	1989.088	0.559	0.894
Classical	Angle-space Ridge	15	2365.950	233.164	1991.072	0.559	0.894
Classical	Ridge (angle-space, tuned)	15	2367.895	236.386	1992.030	0.559	0.894
Classical	Original compact Ridge	15	2368.682	219.562	1991.078	0.557	0.889
Classical	Ridge original plus spd distance (Stein SPD)	15	2377.302	213.438	1999.807	0.553	0.884
Classical	RBF-KRR	15	2382.464	232.329	2003.678	0.553	0.892
Classical	Polynomial-KRR	15	2384.496	233.038	2005.664	0.552	0.894
Classical	Condition means baseline	15	2388.222	214.474	2018.753	0.547	0.856
Classical	LDA condition axis then Ridge raw	15	2395.293	237.229	2007.282	0.548	0.855
Classical	Quadratic Ridge + condition	15	2400.010	229.828	2025.982	0.545	0.883
Classical	Ridge baseline (biomarkers only)	15	2402.836	249.146	2025.918	0.545	0.885
Classical	Ridge + SPD distances (outer-product; best)	15	2406.079	221.828	2031.488	0.542	0.878
Classical	Ridge original plus spd distance (Angular SPD)	15	2409.034	221.510	2022.182	0.542	0.865
Classical	Random forest raw	15	2422.689	239.245	2056.354	0.536	0.873
Classical	Ridge raw	15	2430.492	299.753	2015.455	0.538	0.851
Classical	Random forest engineered	15	2441.953	244.624	2058.497	0.530	0.867
Classical	Shallow decision tree engineered	15	2485.297	238.731	2080.825	0.507	0.847
Classical	Ridge engineered	15	2491.053	311.906	2054.444	0.515	0.859
Classical	Shallow decision tree raw	15	2515.742	236.426	2105.782	0.495	0.852
Classical	SPD-KRR only (Stein SPD)	15	3073.967	381.536	2430.282	0.258	0.805
Classical	SPD-KRR only (Angular SPD)	15	3145.222	426.454	2504.156	0.228	0.791
Classical	Ridge spd distance only (Stein SPD)	15	3229.826	468.222	2537.879	0.186	0.754
Classical	Ridge spd distance only (Angular SPD)	15	3335.313	493.657	2676.394	0.129	0.749
Classical	Global mean baseline	15	3619.197	506.810	2977.348	-0.016	0.500
Quantum	**QKR-full direct compact**	**15**	**2369.948**	**232.293**	**1992.915**	**0.557**	**0.886**
Quantum	QKF-cluster (Nystrom, m=3)	15	2370.610	239.402	1997.355	0.558	0.883
Quantum	SteinDiv-Hilbert-QKR	15	2381.997	205.386	2006.461	0.550	0.877
Quantum	Stein SPD embedded QKR (r=1)	15	2398.519	169.856	2030.747	0.540	0.883
Quantum	Logcosine SPD embedded QKR (r=1)	15	2403.183	168.999	2033.760	0.538	0.873
Quantum	ROSE	15	2408.157	265.347	2020.802	0.543	0.847
Quantum	Synth_ROSE	15	2419.538	229.170	2042.214	0.537	0.849

Lower RMSE and MAE are better; higher R^2 and ROC–AUC are better. Bold horizontal rules mark the best row within each group according to RMSE. Full-row bold marks the overall best RMSE in the table. RMSE is used as the primary ranking criterion. ROSE denotes the mixed-anchor ROSE variant; Synth_ROSE denotes the synthetic-anchor ROSE variant. Only repeated-CV model-family results are used for ranking; exploratory diagnostic runs are not used for main-text ranking.

Table 2: Repeated 3×5 cross-validation main results for muscle quality index.

Group	Model	CV folds	RMSE ↓	RMSE SD	MAE ↓	R^2 ↑	ROC–AUC ↑
Classical	**Original compact Ridge**	**15**	**58.380**	**5.948**	**47.448**	**0.225**	**0.722**
Classical	Biomarker-only Ridge	15	58.470	5.918	47.406	0.223	0.723
Classical	Compact Ridge + condition	15	58.640	5.929	47.570	0.218	0.722
Classical	Ridge original plus spd distance (Angular SPD)	15	58.695	5.635	47.577	0.216	0.715
Classical	Angle-space Ridge	15	58.741	5.969	47.714	0.215	0.719
Classical	Ridge original plus spd distance (Stein SPD)	15	58.742	6.035	47.983	0.215	0.713
Classical	Polynomial-KRR	15	58.829	5.951	47.717	0.213	0.723
Classical	RBF-KRR	15	58.847	5.945	47.726	0.213	0.723
Classical	Quadratic Ridge + condition	15	59.445	5.575	48.246	0.194	0.720
Classical	SPD-KRR only (Stein SPD)	15	62.714	6.165	49.951	0.106	0.678
Classical	Ridge spd distance only (Stein SPD)	15	62.858	6.034	50.351	0.103	0.664
Classical	SPD-KRR only (Angular SPD)	15	63.333	5.380	50.293	0.087	0.664
Classical	Ridge spd distance only (Angular SPD)	15	63.945	6.126	51.253	0.074	0.641
**Quantum**	**Synth_ROSE**	**15**	**58.249**	**5.615**	**47.867**	**0.227**	**0.715**
Quantum	Logcosine SPD embedded VQC (r=1)	15	58.291	5.939	47.791	0.226	0.727
Quantum	ROSE	15	58.504	5.964	47.863	0.217	0.714
Quantum	Stein SPD embedded VQC (r=1)	15	58.716	7.021	48.167	0.215	0.723
Quantum	SteinDiv-Hilbert-QKR	15	58.734	6.051	48.010	0.215	0.713
Quantum	QKR-full direct compact	15	58.885	6.136	48.130	0.208	0.718

Lower RMSE and MAE are better; higher R^2 and ROC–AUC are better. Bold horizontal rules mark the best row within each group according to RMSE. Full-row bold marks the overall best RMSE in the table. RMSE is used as the primary ranking criterion. ROSE denotes the mixed-anchor ROSE variant; Synth_ROSE denotes the synthetic-anchor ROSE variant. Only repeated-CV model-family results are used for ranking; exploratory diagnostic runs are not used for main-text ranking.

Table 3: Repeated 3×5 cross-validation main results for Tibialis Anterior muscle weight.

Group	Model	CV folds	RMSE ↓	RMSE SD	MAE ↓	R^2 ↑	ROC–AUC ↑
Classical	**RBF-KRR**	**15**	**5.210**	**0.770**	**3.432**	**0.473**	**0.786**
Classical	Angle-space Ridge	15	5.231	0.671	3.487	0.469	0.752
Classical	Compact Ridge + condition	15	5.262	0.667	3.520	0.462	0.750
Classical	Polynomial-KRR	15	5.267	0.761	3.475	0.462	0.775
Classical	Original compact Ridge	15	5.289	0.658	3.553	0.456	0.756
Classical	Ridge original plus spd distance (Stein SPD)	15	5.290	0.739	3.557	0.457	0.766
Classical	Biomarker-only Ridge	15	5.368	0.624	3.680	0.439	0.763
Classical	Quadratic Ridge + condition	15	5.397	0.761	3.542	0.432	0.777
Classical	Ridge original plus spd distance (Angular SPD)	15	5.476	0.691	3.707	0.415	0.757
Classical	SPD-KRR only (Stein SPD)	15	6.710	0.741	5.147	0.127	0.677
Classical	Ridge spd distance only (Stein SPD)	15	6.725	0.653	5.387	0.124	0.669
Classical	SPD-KRR only (Angular SPD)	15	6.770	0.781	5.199	0.112	0.675
Classical	Ridge spd distance only (Angular SPD)	15	7.047	0.660	5.666	0.034	0.642
**Quantum**	**Synth_ROSE**	**15**	**5.116**	**0.705**	**3.422**	**0.492**	**0.768**
Quantum	Stein SPD embedded VQC (r=1)	15	5.159	0.645	3.407	0.483	0.780
Quantum	ROSE	15	5.174	0.710	3.448	0.480	0.775
Quantum	QKR-full direct compact	15	5.184	0.714	3.401	0.475	0.788
Quantum	Logcosine SPD embedded VQC (r=1)	15	5.217	0.725	3.448	0.471	0.773
Quantum	SteinDiv-Hilbert-QKR	15	5.506	0.798	3.875	0.415	0.781

Lower RMSE and MAE are better; higher R^2 and ROC–AUC are better. Bold horizontal rules mark the best row within each group according to RMSE. Full-row bold marks the overall best RMSE in the table. RMSE is used as the primary ranking criterion. ROSE denotes the mixed-anchor ROSE variant; Synth_ROSE denotes the synthetic-anchor ROSE variant. Only repeated-CV model-family results are used for ranking; exploratory diagnostic runs are not used for main-text ranking.

Repeated-CV Sensitivity Tables

Table 4: Repeated 3×5 cross-validation sensitivity results for force readout.

Group	Model	CV folds	RMSE ↓	RMSE SD	MAE ↓	R^2 ↑	ROC–AUC ↑
Embedded-r sensitivity	**Stein SPD-kernel-embedded QKR r=1**	**15**	**2398.519**	**169.856**	**2030.747**	**0.540**	**0.883**
Embedded-r sensitivity	Logcosine SPD-kernel-embedded QKR r=1	15	2403.183	168.999	2033.760	0.538	0.873
Embedded-r sensitivity	Logcosine SPD-kernel-embedded QKR r=2	15	2408.498	196.945	2032.486	0.539	0.869
Embedded-r sensitivity	Logcosine SPD-kernel-embedded QKR r=3	15	2415.336	222.646	2031.317	0.538	0.873
Embedded-r sensitivity	Stein SPD-kernel-embedded QKR r=2	15	2415.792	190.534	2039.082	0.534	0.870
Embedded-r sensitivity	Stein SPD-kernel-embedded QKR r=4	15	2421.593	223.997	2040.259	0.536	0.873
Embedded-r sensitivity	Stein SPD-kernel-embedded QKR r=3	15	2429.779	220.725	2042.393	0.531	0.872
Embedded-r sensitivity	Logcosine SPD-kernel-embedded QKR r=4	15	2430.936	228.647	2038.795	0.532	0.863
**ROSE/VQR sensitivity**	**Synth_ROSE tuned**	**15**	**2384.174**	**195.507**	**2007.947**	**0.549**	**0.864**
ROSE/VQR sensitivity	VQR-compact	15	2409.215	214.107	2028.180	0.539	0.854
ROSE/VQR sensitivity	SteinDiv-Hilbert-QKR	15	2416.403	176.537	2040.126	0.533	0.868
Real-to-synthetic sensitivity	**RTS-SteinDist-RP-QKR syn=0.5**	**15**	**2393.841**	**250.894**	**2009.317**	**0.549**	**0.863**
Real-to-synthetic sensitivity	RTS-SteinDist-RP-QKR syn=1	15	2413.972	220.043	2025.096	0.538	0.841
Real-to-synthetic sensitivity	RTS-SteinKernel-RP-VQR syn=0.5	15	2417.096	225.808	2036.015	0.537	0.855
Real-to-synthetic sensitivity	RTS Synth_ROSE	15	2422.532	220.853	2038.847	0.533	0.858
Real-to-synthetic sensitivity	RTS-SteinKernel-RP-VQR syn=1	15	2425.681	205.739	2030.100	0.532	0.844
Real-to-synthetic sensitivity	RTS-SteinDist-RP-QKR	15	2429.511	180.545	2047.337	0.527	0.863

Lower RMSE and MAE are better; higher R^2 and ROC–AUC are better. Bold horizontal rules mark the best row within each group according to RMSE. Full-row bold marks the overall best RMSE in the table. RMSE is used as the primary ranking criterion. Exploratory stacking, endpoint-blend, and calibration analyses were treated as diagnostic sensitivity analyses and were not used for formal model ranking.

Table 5: Repeated 3×5 cross-validation sensitivity results for muscle quality index.

Group	Model	CV folds	RMSE ↓	RMSE SD	MAE ↓	R^2 ↑	ROC–AUC ↑
**Embedded-r sensitivity**	**Logcosine SPD-kernel-embedded VQC r=1**	**15**	**58.291**	**5.939**	**47.791**	**0.226**	**0.727**
Embedded-r sensitivity	Stein SPD-kernel-embedded VQC r=1	15	58.716	7.021	48.167	0.215	0.723
Embedded-r sensitivity	Stein SPD-kernel-embedded VQC r=4	15	59.245	5.597	48.471	0.196	0.704
Embedded-r sensitivity	Stein SPD-kernel-embedded VQC r=2	15	59.261	6.048	48.617	0.200	0.691
Embedded-r sensitivity	Logcosine SPD-kernel-embedded VQC r=2	15	59.305	6.115	48.524	0.198	0.700
Embedded-r sensitivity	Logcosine SPD-kernel-embedded VQC r=3	15	59.663	5.334	48.505	0.188	0.691
Embedded-r sensitivity	Stein SPD-kernel-embedded VQC r=3	15	59.842	6.536	48.576	0.182	0.690
Embedded-r sensitivity	Logcosine SPD-kernel-embedded VQC r=4	15	59.946	5.936	48.860	0.180	0.686
ROSE/VQR sensitivity	**VQR-compact**	**15**	**58.805**	**5.550**	**47.942**	**0.211**	**0.721**
ROSE/VQR sensitivity	SteinDiv-Hilbert-QKR	15	58.940	6.577	48.149	0.210	0.699
ROSE/VQR sensitivity	Synth_ROSE tuned	15	59.136	6.672	48.195	0.208	0.704
Real-to-synthetic sensitivity	**RTS-SteinDist-RP-QKR syn=0.5**	**15**	**58.499**	**6.280**	**47.909**	**0.221**	**0.693**
Real-to-synthetic sensitivity	RTS-SteinDist-RP-QKR	15	58.633	6.305	47.926	0.219	0.704
Real-to-synthetic sensitivity	RTS Synth_ROSE	15	58.682	6.355	48.074	0.217	0.691
Real-to-synthetic sensitivity	RTS-SteinKernel-RP-VQR syn=0.5	15	58.708	6.217	48.206	0.217	0.715

Continued on next page

Table 5: Repeated 3×5 cross-validation sensitivity results for muscle quality index. (continued)

Group	Model	CV folds	RMSE ↓	RMSE SD	MAE ↓	R^2 ↑	ROC–AUC ↑
Real-to-synthetic sensitivity	RTS-SteinDist-RP-QKR syn=1	15	58.806	6.238	47.926	0.213	0.694
Real-to-synthetic sensitivity	RTS-SteinKernel-RP-VQR syn=1	15	59.357	6.631	48.465	0.199	0.717

Lower RMSE and MAE are better; higher R^2 and ROC–AUC are better. Bold horizontal rules mark the best row within each group according to RMSE. Full-row bold marks the overall best RMSE in the table. RMSE is used as the primary ranking criterion. Exploratory stacking, endpoint-blend, and calibration analyses were treated as diagnostic sensitivity analyses and were not used for formal model ranking.

Table 6: Repeated 3×5 cross-validation sensitivity results for Tibialis Anterior muscle weight.

Group	Model	CV folds	RMSE ↓	RMSE SD	MAE ↓	R^2 ↑	ROC–AUC ↑
Embedded-r sensitivity	**Stein SPD-kernel-embedded VQC r=1**	**15**	**5.159**	**0.645**	**3.407**	**0.483**	**0.780**
Embedded-r sensitivity	Stein SPD-kernel-embedded VQC r=2	15	5.186	0.641	3.481	0.475	0.781
Embedded-r sensitivity	Logcosine SPD-kernel-embedded VQC r=1	15	5.217	0.725	3.448	0.471	0.773
Embedded-r sensitivity	Logcosine SPD-kernel-embedded VQC r=2	15	5.224	0.784	3.478	0.470	0.773
Embedded-r sensitivity	Logcosine SPD-kernel-embedded VQC r=3	15	5.225	0.670	3.575	0.469	0.798
Embedded-r sensitivity	Stein SPD-kernel-embedded VQC r=3	15	5.227	0.613	3.501	0.465	0.782
Embedded-r sensitivity	Stein SPD-kernel-embedded VQC r=4	15	5.235	0.690	3.577	0.468	0.789
Embedded-r sensitivity	Logcosine SPD-kernel-embedded VQC r=4	15	5.266	0.696	3.528	0.464	0.798
ROSE/VQR sensitivity	**Synth_ROSE tuned**	**15**	**5.143**	**0.698**	**3.430**	**0.486**	**0.786**
ROSE/VQR sensitivity	VQR-compact	15	5.219	0.694	3.500	0.470	0.787
ROSE/VQR sensitivity	SteinDiv-Hilbert-QKR	15	5.366	0.798	3.596	0.441	0.787
**Real-to-synthetic sensitivity**	**RTS Synth_ROSE**	**15**	**5.134**	**0.716**	**3.429**	**0.490**	**0.779**
Real-to-synthetic sensitivity	RTS-SteinKernel-RP-VQR syn=1	15	5.181	0.688	3.459	0.480	0.767
Real-to-synthetic sensitivity	RTS-SteinKernel-RP-VQR syn=0.5	15	5.247	0.774	3.497	0.464	0.767
Real-to-synthetic sensitivity	RTS-SteinDist-RP-QKR	15	5.255	0.773	3.485	0.462	0.784
Real-to-synthetic sensitivity	RTS-SteinDist-RP-QKR syn=1	15	5.653	0.808	3.975	0.382	0.787
Real-to-synthetic sensitivity	RTS-SteinDist-RP-QKR syn=0.5	15	5.939	0.818	4.110	0.319	0.766

Lower RMSE and MAE are better; higher R^2 and ROC–AUC are better. Bold horizontal rules mark the best row within each group according to RMSE. Full-row bold marks the overall best RMSE in the table. RMSE is used as the primary ranking criterion. Exploratory stacking, endpoint-blend, and calibration analyses were treated as diagnostic sensitivity analyses and were not used for formal model ranking.

Paired repeated-CV statistical tests

Paired repeated-CV statistical comparison. The repeated-CV summaries report the mean and standard deviation of each metric across the 15 held-out folds obtained from three repeats of five-fold cross-validation. For statistical comparison, models were compared in a paired manner at the outer-fold level: for each endpoint and each split, the metric from the best quantum model was paired with the metric from the best classical model evaluated on the corresponding held-out fold. The primary comparison used the fold-wise RMSE difference, defined as $\Delta_i = \mathrm{RMSE}_{\mathrm{quantum},i} - \mathrm{RMSE}_{\mathrm{classical},i}$, so that negative values favour the quantum model. Because the number of fold pairs was small ($n = 15$) and normality of fold-wise differences should not be assumed, the Wilcoxon signed-rank test was used as the primary non-parametric paired test. Holm adjustment was applied across the three endpoint-level primary RMSE comparisons. A corrected repeated-CV paired t-test was also calculated as a secondary sensitivity statistic, using the repeated-CV correction for the variance of fold-wise differences. The Wilcoxon test is the primary reported test because it is less sensitive to distributional assumptions.

Sensitivity testing. The same fold-wise paired procedure was repeated for each quantum sensitivity variant against the best classical model for the corresponding endpoint. Holm correction was applied within each endpoint for the sensitivity comparisons.

Table 7: Primary paired repeated-CV RMSE comparison between the best quantum and best classical model for each endpoint. The difference is quantum minus classical; negative values favour the quantum model.

Task	Best classical	Best quantum	Classical	Quantum	Δ	Wilcoxon p	Holm p
Force	Biomarker-only Ridge	QKR-full direct compact	2358.459	2369.948	11.489	0.890	1.000
Quality	Original compact Ridge	Synth_ROSE	58.380	58.249	-0.131	0.762	1.000
Weight	RBF-KRR	Synth_ROSE	5.210	5.116	-0.094	0.095	0.284

Table 8: Wilcoxon signed-rank RMSE sensitivity analysis for force. Each quantum sensitivity variant is compared with the best classical model for the endpoint.

Quantum sensitivity model	Classical RMSE	Quantum RMSE	ΔRMSE	Wilcoxon p	Holm p
Synth_ROSE tuned	2358.459	2384.174	25.715	0.169	0.554
RTS-SteinDist-RP-QKR syn=0.5	2358.459	2393.841	35.382	0.055	0.554
Stein SPD-kernel-embedded QKR r=1	2358.459	2398.519	40.061	0.229	0.554
Logcosine SPD-kernel-embedded QKR r=1	2358.459	2403.183	44.724	0.169	0.554
Logcosine SPD-kernel-embedded QKR r=2	2358.459	2408.498	50.039	0.064	0.554
VQR-compact	2358.459	2409.215	50.756	0.055	0.554
RTS-SteinDist-RP-QKR syn=1	2358.459	2413.972	55.514	0.012	0.187
Logcosine SPD-kernel-embedded QKR r=3	2358.459	2415.336	56.877	0.026	0.332
Stein SPD-kernel-embedded QKR r=2	2358.459	2415.792	57.333	0.064	0.554
SteinDiv-Hilbert-QKR	2358.459	2416.403	57.944	0.107	0.554
RTS-SteinKernel-RP-VQR syn=0.5	2358.459	2417.096	58.637	0.064	0.554
Stein SPD-kernel-embedded QKR r=4	2358.459	2421.593	63.134	0.010	0.164
RTS Synth_ROSE	2358.459	2422.532	64.073	0.048	0.527
RTS-SteinKernel-RP-VQR syn=1	2358.459	2425.681	67.223	0.041	0.495
RTS-SteinDist-RP-QKR	2358.459	2429.511	71.052	0.055	0.554
Stein SPD-kernel-embedded QKR r=3	2358.459	2429.779	71.320	0.022	0.302
Logcosine SPD-kernel-embedded QKR r=4	2358.459	2430.936	72.477	0.007	0.114

Table 9: Wilcoxon signed-rank RMSE sensitivity analysis for quality. Each quantum sensitivity variant is compared with the best classical model for the endpoint.

Quantum sensitivity model	Classical RMSE	Quantum RMSE	ΔRMSE	Wilcoxon p	Holm p
**Logcosine SPD-kernel-embedded VQC r=1**	58.380	**58.291**	**-0.089**	0.978	1.000
RTS-SteinDist-RP-QKR syn=0.5	58.380	58.499	0.119	0.934	1.000
RTS-SteinDist-RP-QKR	58.380	58.633	0.253	0.359	1.000
RTS Synth_ROSE	58.380	58.682	0.302	0.229	1.000
RTS-SteinKernel-RP-VQR syn=0.5	58.380	58.708	0.328	0.277	1.000
Stein SPD-kernel-embedded VQC r=1	58.380	58.716	0.337	0.934	1.000
VQR-compact	58.380	58.805	0.426	0.169	1.000
RTS-SteinDist-RP-QKR syn=1	58.380	58.806	0.427	0.330	1.000
SteinDiv-Hilbert-QKR	58.380	58.940	0.560	0.135	1.000
Synth_ROSE tuned	58.380	59.136	0.756	0.135	1.000
Stein SPD-kernel-embedded VQC r=4	58.380	59.245	0.865	0.107	1.000
Stein SPD-kernel-embedded VQC r=2	58.380	59.261	0.882	0.022	0.280
Logcosine SPD-kernel-embedded VQC r=2	58.380	59.305	0.925	0.018	0.253
RTS-SteinKernel-RP-VQR syn=1	58.380	59.357	0.977	0.135	1.000
Logcosine SPD-kernel-embedded VQC r=3	58.380	59.663	1.283	0.003	0.042
Stein SPD-kernel-embedded VQC r=3	58.380	59.842	1.463	0.015	0.226
Logcosine SPD-kernel-embedded VQC r=4	58.380	59.946	1.566	< 0.001	0.010

Table 10: Wilcoxon signed-rank RMSE sensitivity analysis for weight. Each quantum sensitivity variant is compared with the best classical model for the endpoint.

Quantum sensitivity model	Classical RMSE	Quantum RMSE	ΔRMSE	Wilcoxon p	Holm p
**RTS Synth_ROSE**	5.210	**5.134**	**-0.076**	0.330	1.000
**Synth_ROSE tuned**	5.210	**5.143**	**-0.067**	0.252	1.000
**Stein SPD-kernel-embedded VQC r=1**	5.210	**5.159**	**-0.051**	0.561	1.000
**RTS-SteinKernel-RP-VQR syn=1**	5.210	**5.181**	**-0.029**	0.599	1.000
**Stein SPD-kernel-embedded VQC r=2**	5.210	**5.186**	**-0.023**	0.599	1.000
Logcosine SPD-kernel-embedded VQC r=1	5.210	5.217	0.007	0.890	1.000
VQR-compact	5.210	5.219	0.009	0.890	1.000
Logcosine SPD-kernel-embedded VQC r=2	5.210	5.224	0.014	0.978	1.000
Logcosine SPD-kernel-embedded VQC r=3	5.210	5.225	0.015	0.762	1.000
Stein SPD-kernel-embedded VQC r=3	5.210	5.227	0.017	0.978	1.000
Stein SPD-kernel-embedded VQC r=4	5.210	5.235	0.026	0.934	1.000
RTS-SteinKernel-RP-VQR syn=0.5	5.210	5.247	0.038	0.679	1.000
RTS-SteinDist-RP-QKR	5.210	5.255	0.045	0.599	1.000
Logcosine SPD-kernel-embedded VQC r=4	5.210	5.266	0.056	0.489	1.000
SteinDiv-Hilbert-QKR	5.210	5.366	0.157	0.151	1.000
RTS-SteinDist-RP-QKR syn=1	5.210	5.653	0.443	< 0.001	0.007
RTS-SteinDist-RP-QKR syn=0.5	5.210	5.939	0.730	< 0.001	0.002

Synthetic-medoid Stein–Nystrom QKR audit

This supplementary audit compares uniformly sampled synthetic references with synthetic-only k-medoid references for the Stein–Nystrom QKR branch. It is included as a reference-selection sensitivity analysis and is not used to replace the main endpoint rankings.

Table 11: Synthetic-medoid versus random synthetic-reference summary.

Task	Reference mode	CV folds	RMSE	MAE	ROC–AUC
Force	medoids	15	2423.001	2023.675	0.851
Force	random	15	2427.768	2024.348	0.856
Quality	random	15	59.257	48.056	0.707
Quality	medoids	15	59.364	47.846	0.708
Weight	random	15	5.185	3.427	0.794
Weight	medoids	15	5.197	3.435	0.793

Table 12: Corrected paired RMSE comparison for synthetic-medoid versus random synthetic references. Negative differences favour medoids.

Task	Medoid RMSE	Random RMSE	Δ	Corrected p
Force	2423.001	2427.768	-4.767	0.865
Quality	59.364	59.257	0.107	0.866
Weight	5.197	5.185	0.011	0.919

0.1 Compared regression and quantum-kernel models

All models were evaluated using the same repeated cross-validation protocol used for the performance tables. The outer evaluation used repeated stratified five-fold cross-validation with three repeats, giving 15 held-out folds per endpoint. Stratification was performed with respect to experimental condition. All preprocessing operations, feature transformations, synthetic SPD generation, prototype or landmark selection, kernel scaling, angle normalisation, and hyperparameter selection were performed inside the corresponding outer-training fold only. Tunable hyperparameters were selected by inner cross-validation within the outer-training data, and the held-out fold was used only for held-out evaluation. Model selection in the tables was based primarily on held-out RMSE, with MAE, R^2, and ROC-AUC reported as complementary metrics.

0.1.1 Classical baselines

The global-mean baseline predicts the mean target value estimated from the outer-training fold. The condition-mean baseline predicts the training-fold mean corresponding to the observed experimental condition of each held-out sample. These two baselines provide lower-bound references for models that either ignore covariates entirely or use only the group-level condition signal.

The raw-feature Ridge model fits an ℓ_2-regularised linear regression model to the available non-target biological descriptors. Missing values are imputed using the training-fold median, the features are standardised using training-fold statistics, and the Ridge penalty is selected by inner cross-validation. The engineered Ridge model uses the same training-only imputation and standardisation but additionally applies a Yeo–Johnson power transformation and a second-order polynomial expansion before fitting Ridge regression. Thus, the engineered model tests whether simple nonlinear feature interactions improve prediction without using quantum or SPD representations.

The random forest and shallow decision-tree regressors are conventional nonlinear tree-based baselines. They are trained on either the raw or engineered feature sets after the same training-fold preprocessing. Their hyperparameters, including tree depth, leaf-size constraints, and related regularisation settings, are selected within the inner cross-validation loop. These models test whether improvements can be explained by generic nonlinear tabular learning rather than by the proposed SPD or quantum representations.

The LDA-condition-axis Ridge model first learns a single supervised axis that separates the experimental conditions in the outer-training fold using linear discriminant analysis. The resulting one-dimensional condition-discriminative coordinate is then combined with the observed condition indicator and fitted with Ridge regression. This baseline tests whether a low-dimensional classical condition axis is sufficient to capture the information used by the more structured models.

The biomarker-only Ridge baseline uses only the endpoint-specific compact biomarker set selected for each target. For weight, the compact features are blood CRP, neutrophils, and total BALF cell count. For force, the compact features are muscle oxidative stress, lung TNFα mRNA, and neutrophils. For muscle quality, the compact features are lung TNFα mRNA, neutrophils, and lymphocytes. This baseline is important because it controls for the strongest compact non-quantum representation used by several quantum and SPD models.

The angle-space Ridge model applies the same compact-feature preprocessing used before quantum angle encoding, but instead of evaluating a quantum kernel it fits Ridge regression directly in the angle-normalised feature space. Specifically, compact inputs are imputed, transformed, standardised, optionally reduced to the effective qubit dimension, and mapped into $[-\pi/2, \pi/2]$ using training-fold min–max scaling. This model tests whether performance gains are due merely to the angle-space normalisation rather than to the quantum kernel itself.

Polynomial-KRR and RBF-KRR are classical kernel-ridge baselines fitted to the compact descriptors. Polynomial-KRR uses a polynomial kernel, while RBF-KRR uses a radial-basis-function kernel. These baselines provide direct classical-kernel comparators for quantum-kernel ridge regression under the same repeated-CV protocol.

0.1.2 SPD-distance classical models

The SPD-distance Ridge models convert each compact feature vector into an SPD descriptor and then use distances to train-fold SPD prototypes as additional Ridge-regression features. In the outer-product version, a standardised vector x is normalised and embedded as

$$S(x) = \frac{xx^\top}{\|x\|_2^2} + \epsilon I, \tag{1}$$

where ϵI ensures positive definiteness. In the full-rank SPD extension, each sample is represented by a regularised neighbourhood covariance matrix estimated from its nearest training-fold neighbours, giving a local SPD descriptor rather than a rank-one descriptor. Test samples are described only with respect to training-fold neighbours, so the held-out data do not influence the SPD representation.

Distances between SPD descriptors are computed using either the Stein divergence or a log-Euclidean angular/log-cosine distance. The Stein divergence between SPD matrices A and B is

$$D_{\text{Stein}}(A, B) = \log\det\left(\frac{A + B}{2}\right) - \frac{1}{2}\log\det(A) - \frac{1}{2}\log\det(B). \tag{2}$$

The angular SPD distance is computed after mapping SPD matrices to the tangent/log domain, vectorising the matrix logarithms, and comparing their cosine similarity. Train-fold medoids or prototypes are selected using only the training SPD distance matrix. Ridge regression is then fitted either on the SPD-distance features alone or on the concatenation of the original compact descriptors and the SPD-distance features.

The SPD-KRR-only models use the SPD-derived kernel directly in kernel ridge regression. The SPD kernel has the form

$$K_{\text{SPD}}(A, B) = \exp\{-\gamma D_{\text{SPD}}(A, B)\}, \tag{3}$$

where D_{SPD} is either the Stein or angular/log-cosine SPD distance. The kernel bandwidth γ is set from the median positive training-fold SPD distance, optionally multiplied by a tuning factor. Kernel construction, bandwidth estimation, and kernel normalisation are all restricted to the training fold.

0.1.3 Quantum-kernel and quantum-feature models

The direct QKR model applies a train-only angle encoding to the compact descriptors and evaluates a fidelity quantum kernel. After imputation, power transformation, standardisation, and optional PCA to the effective qubit dimension, each feature vector is scaled to an angle vector. A shallow circuit applies data-dependent R_y and R_z rotations followed by a nearest-neighbour CNOT entangling chain. The quantum kernel between two encoded samples is the squared state fidelity,

$$K_Q(x_i, x_j) = |\langle\psi(x_i) \mid \psi(x_j)\rangle|^2, \tag{4}$$

optionally transformed by a kernel-power parameter. The final continuous prediction is obtained using kernel ridge regression with this precomputed quantum kernel.

The QKF-cluster model is a compact Nyström approximation to the full quantum kernel. Training-fold samples are first mapped into the same angle-normalised space. K-means is then used inside the training fold to select a small number of quantum feature centres. Each sample is represented by its fidelity-kernel similarities to these centres; the centre-centre kernel is whitened using a Nyström correction. Ridge regression is fitted to the resulting low-dimensional quantum feature map. This model tests whether most of the useful quantum-kernel signal can be retained by a small set of train-only representative centres.

The VQR-compact model uses the compact biomarker representation, concatenated with condition where specified by the code, and maps it through a shallow variational quantum readout. The circuit uses train-only angle normalisation followed by layers of R_y/R_z rotations and CNOT entanglement. Rather than using the full pairwise quantum kernel, it extracts measurement-derived Z-expectation features from the circuit. A Ridge regression head is then fitted to these quantum readout features to predict the continuous endpoint. Random variational parameters are selected using only the fitting data, and the Ridge head is trained on the outer-training fold.

The SPD-kernel-embedded QKR/VQC models first map the compact descriptors to SPD matrices and then form an SPD kernel or SPD distance representation using either Stein or log-cosine geometry. The SPD kernel is then embedded into a low-dimensional coordinate system with dimension r. For force, the embedded coordinates are passed to QKR, producing a fidelity kernel followed by kernel ridge regression. For weight and quality, the embedded coordinates are passed through a VQC-style quantum readout, and the resulting quantum measurement features are used by a Ridge regression head for the continuous endpoint. The reported sensitivity over $r = 1, 2, 3, 4$ examines how much SPD-kernel embedding dimension is needed.

0.1.4 ROSE, Synth_ROSE, and Stein-divergence Hilbert bridge models

The ROSE-family models construct a train-only SPD representation, generate synthetic SPD matrices inside the outer-training fold, and use the Stein kernel to project the data into a randomised RKHS coordinate system. Synthetic SPD matrices are generated in the log-Euclidean tangent space by interpolation or mild extrapolation between training SPD matrices, with small tangent noise and clipping to a training-derived log-ball. This prevents synthetic samples from being generated outside the range supported by the training fold.

In the original ROSE model, the reference landmark pool can include both real training SPD matrices and synthetic SPD matrices. In Synth_ROSE, the synthetic-anchor variant, the random reference anchors used to form the Stein-kernel distance or similarity representation are selected from the synthetic SPD pool. This distinction is important because Synth_ROSE tests whether real samples can be represented through synthetic SPD anchors rather than through a mixed real–synthetic reference set.

For ROSE/Synth_ROSE, the Stein kernel on the reference set is whitened using an inverse square-root kernel factor. Random projection directions are generated by contrasting a small random subset of reference points against the reference-set mean. The projected RKHS features are then angle-normalised and passed into a shallow VQR/VQC-style quantum readout, followed by a Ridge regression head for the continuous endpoint. This produces an SPD-normalised quantum readout model whose synthetic generation, kernel whitening, projection, angle scaling, and Ridge fitting are all repeated independently inside each training fold.

The SteinDiv-Hilbert-QKR model uses the Stein divergence as a distance rather than first forming a random RKHS projection. A train-only reference set of real and/or synthetic SPD matrices is built, and the Stein-divergence matrix is double-centred to form a Hilbert/MDS coordinate representation:

$$G = -\frac{1}{2} H D_{\text{Stein}} H, \qquad H = I - \frac{1}{n}\mathbf{1}\mathbf{1}^\top. \tag{5}$$

The leading positive eigen-directions provide the retained coordinates. These coordinates are then angle-normalised and passed to QKR, where the final prediction is made by kernel ridge regression on the quantum fidelity kernel.

0.1.5 Real-to-synthetic sensitivity models

The real-to-synthetic sensitivity models enforce a stricter audit condition: real training and held-out samples are represented only by their distances or kernel similarities to synthetic SPD matrices generated inside the corresponding outer-training fold. No real–real distance matrix is used as the final feature map, and no mixed real–synthetic landmark pool is used for the reported representation. In the RTS-SteinDist-RP-QKR variant, Stein-divergence distances from real samples to synthetic SPD references are randomly projected and then used by QKR. In the RTS-SteinKernel-RP-VQR variant, Stein-kernel similarities to synthetic SPD references are randomly projected and passed through a VQR-style quantum readout. The synthetic multiplier controls the number of synthetic SPD references relative to the number of outer-training samples.
